# Attention-optimized DeepLab V3 + for automatic estimation of cucumber disease severity

**DOI:** 10.1186/s13007-022-00941-8

**Published:** 2022-09-06

**Authors:** Kaiyu Li, Lingxian Zhang, Bo Li, Shufei Li, Juncheng Ma

**Affiliations:** 1 College of Information and Electrical Engineering, China Agricultural University, Beijing, 100083 China; 2 Key Laboratory of Agricultural Informationization Standardization, Ministry of Agriculture and Rural Affairs, Beijing, 100083 China; 3 Institute of Environment and Sustainable Development in Agriculture, Chinese Academy of Agricultural Sciences, Beijing, 100081 China; 4 Beijing Information Science and Technology University, 100192, Beijing, China

**Keywords:** Semantic segmentation, DeepLab V3 +, Attention mechanism, Transfer learning, Disease severity

## Abstract

**Background:**

Automatic and accurate estimation of disease severity is critical for disease management and yield loss prediction. Conventional disease severity estimation is performed using images with simple backgrounds, which is limited in practical applications. Thus, there is an urgent need to develop a method for estimating the disease severity of plants based on leaf images captured in field conditions, which is very challenging since the intensity of sunlight is constantly changing, and the image background is complicated.

**Results:**

This study developed a simple and accurate image-based disease severity estimation method using an optimized neural network. A hybrid attention and transfer learning optimized semantic segmentation model was proposed to obtain the disease segmentation map. The severity was calculated by the ratio of lesion pixels to leaf pixels. The proposed method was validated using cucumber downy mildew, and powdery mildew leaves collected under natural conditions. The results showed that hybrid attention with the interaction of spatial attention and channel attention can extract fine lesion and leaf features, and transfer learning can further improve the segmentation accuracy of the model. The proposed method can accurately segment healthy leaves and lesions (MIoU = 81.23%, FWIoU = 91.89%). In addition, the severity of cucumber leaf disease was accurately estimated (R^2^ = 0.9578, RMSE = 1.1385). Moreover, the proposed model was compared with six different backbones and four semantic segmentation models. The results show that the proposed model outperforms the compared models under complex conditions, and can refine lesion segmentation and accurately estimate the disease severity.

**Conclusions:**

The proposed method was an efficient tool for disease severity estimation in field conditions. This study can facilitate the implementation of artificial intelligence for rapid disease severity estimation and control in agriculture.

## Background

Cucumber (Cucumis sativus L.) is the world's third most consumed vegetable crop, with an annual production of over 83 million metric tons [1]. Disease is one of the leading causes that decrease cucumber quality and cause economic losses to farmers, especially when cucumber is highly susceptible to downy mildew and powdery mildew. The disease severity is crucial in defining treatment plans and predicting crop loss. Accurate quantification of disease severity helps evaluate the efficiency of disease control measures [2]. Therefore, it is essential to conduct disease severity estimation.

Visual estimation is the conventional approach to quantify the disease severity, which assign a severity value to the symptoms perceived by the human eye in the visible light range. Disease severity based on ratio scales is usually performed by manual estimation of the visual score according to the number and the area of the plants’ lesions [3]. It has been proved the most accurate tool for estimating the disease severity [4]. However, the visual estimation is not robust due to the heterogeneity of different disease symptoms and the subjective nature. Severity assessment based on digital image analysis can be accurate and repeatable, which is therefore widely used for disease severity assessment and consists of a similar process. First, background noises are eliminated using image preprocessing or through a manual operation [5]. Many researchers have combined color transformation with mathematical morphology operations [6], thresholding [7], and filtering [8] to achieve lesion segmentation. These algorithms are quick, easy to develop, and simple to implement under controlled conditions. However, disease images collected in field conditions contain a lot of noise, such as illumination and cluttered backgrounds, and the features are diverse and complex. Therefore, these methods tend to be limited in segmenting lesions in field conditions since they mainly rely on the manually-designed image features.

Deep Learning (DL) has emerged as the state-of-the-art image processing technology, which can perform automatically-feature learning. The DL technology is widely used in medical segmentation [9], road detection [10], and disease diagnosis [2, 11–13], and has achieved satisfactory results. For the DL-based disease severity estimation, the methods can be roughly classified into three categories: classification-based, regression-based, and semantic segmentation-based.

The classification-based methods adopt convolutional neural networks (CNNs) and transform it into a classification problem by defining the severity categories or intervals. Wang et al. classified the black rot severity into four categories: healthy, early stage, middle stage, and late stage [14]. The authors trained the VGG16 network by transfer learning and achieved an accuracy of over 90%. Liang et al. proposed a deep learning method using ResNet50 as the base model and shuffle units as the auxiliary structure [15]. The method classified the disease severity into three categories (healthy, general, and severe type), achieving an overall accuracy of 91%. Esgario et al. used a similar classification-based method for severity estimation [16]. The accuracy for the five severity categories that were defined in this method was 86.51%. Although accurate results were reported in the above studies, dividing severity percentages into multiple categories in field trials did not make it easy to assess the effectiveness of treatments, such as fungicides [3].

On the contrary, the regression-based and semantic segmentation-based methods can yield the severity percentage, which is more informative. Zhang et al. constructed a CNN model taking background-removed cucumber leaf images as input and the severity of cucumber downy mildew as output, achieving an *R*^2^ of 0.9190 [17]. However, we tested this model on a dataset of cucumber leaves with complex backgrounds, finding that it failed to accurately estimate severity. Semantic segmentation has achieved remarkable results in crop segmentation [2, 18] and disease lesion segmentation [19, 20]. Lin et al. achieved pixel-level segmentation of cucumber powdery mildew using the Unet with an average pixel accuracy of 96.08% [20]. After removing the backgrounds from the tomato disease images, Wspanialy et al. also used the Unet for disease lesion segmentation [19]. The error of the severity estimation was 11.8%. These studies provide support for disease severity by semantic segmentation. Gonçalves et al. applied multiple semantic segmentation methods to laboratory-acquired images to assess severity [21]. The results showed that DeepLab V3 + performs better in severity estimation. However, images taken in field conditions will inevitably have cluttered backgrounds, thus reducing the severity estimation accuracy [22]. In light of these publications and results, Wang et al. designed a disease segmentation model with a two-stage architecture [22]. In this model, the cucumber leaves and disease lesions are sequentially segmented. The severity of downy mildew is then classified based on the segmentation results, reducing the influence of complex backgrounds. However, this two-stage segmentation approach costs more computing resources and increases the complexity of the severity estimation task. Therefore, exploring a straightforward, suitable, and efficient severity estimation method for cucumber disease images in field conditions is necessary.

In this study, an optimized DeepLab V3 + [23] segmentation model is proposed to automatically estimate the severity of cucumber leaf diseases in field conditions, i.e., cucumber downy mildew, and powdery mildew. The residual network is used for feature extraction and the hybrid attention is incorporated to suppress background information and improve the ability to express lesion features. Transfer learning is adopted to improve segmentation accuracy. Compared with the existing methods, the proposed method has three significant contributions that are summarized as follows:

- A pixel-level classification-based method is proposed for direct and automatic severity estimation of cucumber downy mildew and powdery mildew using images with complex backgrounds. By calculating the ratio of the lesion area over the leaf area, this method can accurately estimate the cucumber disease severity.
- A segmentation model based on a fine-tuned DeepLab V3 + and a hybrid attention mechanism is proposed to improve the model’s ability to express lesions features. The model reduces the influence of complex backgrounds on the recognition performance of lesions and healthy leaves and achieves accurate lesion segmentation.
- Comparison between the proposed method and the widely used attention mechanisms, backbones, and semantic segmentation models are performed. The relationship between the severity estimated by models and manual visual scoring is then quantified.

## Materials and methods

### Image acquisition and preprocessing

We collected the image data in the No. 5 daylight greenhouse at the Agricultural Innovation Base of Tianjin Academy of Agricultural Sciences. A total of 153 images were collected from 8:00 to 17:00 on April 20, 2016, using a Nikon Coolpix S3100 digital camera in automatic mode. We did not use optical zoom or flash during the image acquisition. Image preprocessing was used to reduce computational costs and improve computing efficiency. Specifically, the image size was uniformly adjusted to 224 × 224 pixels.

**Fig. 1:**
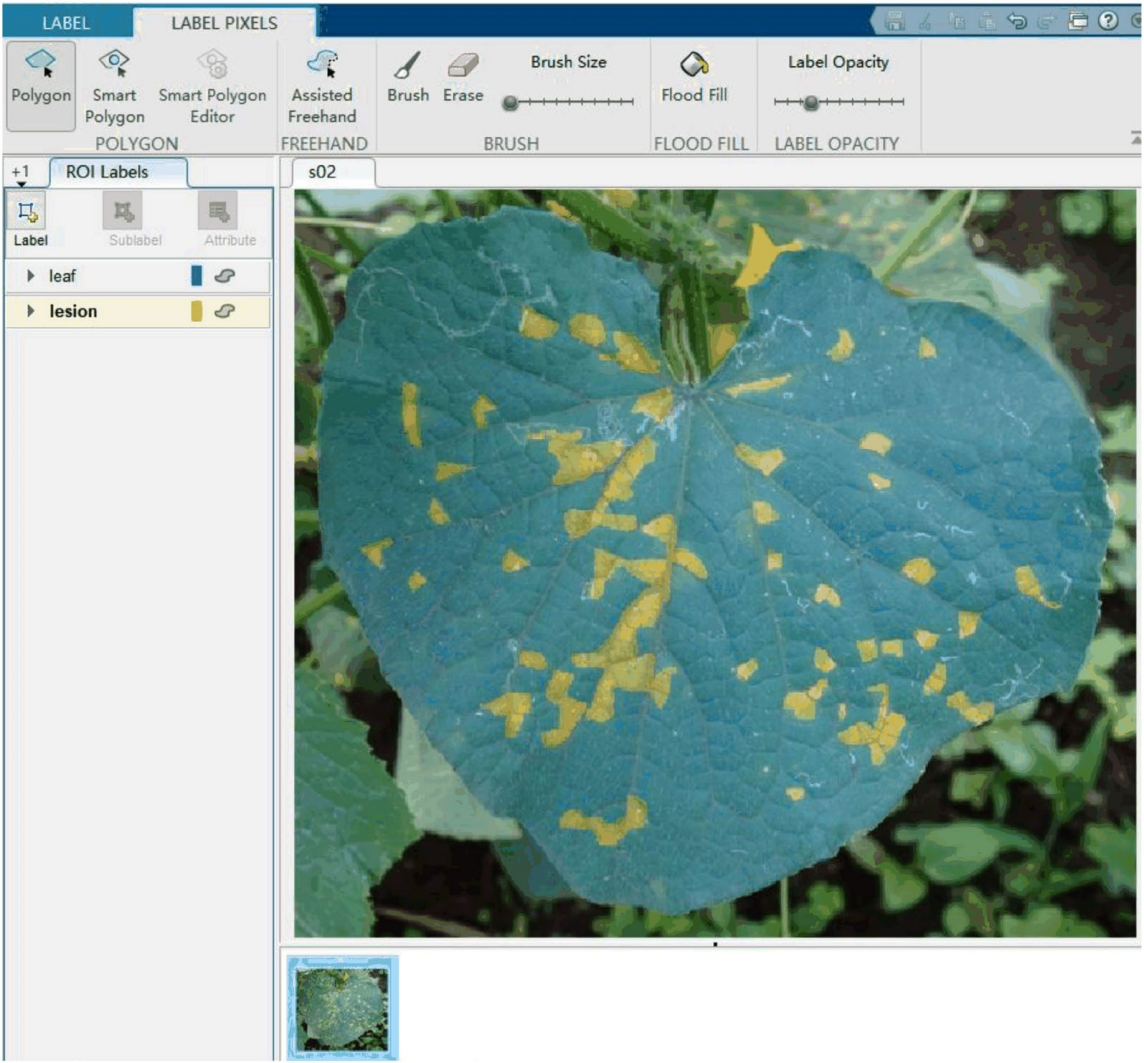
Image annotation process. Blue and yellow pixels indicate the leaf and the lesion, respectively. The rest of the image is the background

The pixel-wise annotation of the diseased images is performed using MATLAB Image Labeler App (MathWorks Inc., USA). The annotation process is shown in Fig. 1. During the image labeling, there might be more than one leaf in an image. Therefore, image labeling is performed only on the leaf at the center. The background, leaf, and disease lesion categories are marked as 0, 1, and 2, respectively. The severity of the disease is computed as:

1 $$\mathrm{Disease Severity}=\frac{{\mathrm{P}}_{\mathrm{lesion}}}{{\mathrm{P}}_{\mathrm{lesion}}+{\mathrm{P}}_{\mathrm{leaf}}}$$

where, ${\mathrm{P}}_{\mathrm{lesion}}$ is the number of pixels of the lesions, and ${\mathrm{P}}_{\mathrm{leaf}}$ is the number of pixels of the healthy leaves in the image.

The dataset used in this study consists of 76 downy mildew images and 77 powdery mildew images, which is then divided into training, validation, and test sub-datasets by following the ratio of 6:2:2 based on stratified sampling. Since there are only 93 images in the training dataset, data augmentation is performed for each disease category to prevent overfitting and improve the generalization ability. The augmentation strategies consist of horizontal and vertical flip, random scaling, clockwise and counterclockwise rotation of the original images by 90°. Consequently, the number of images in the training, validation, and test datasets is 2976, 30, and 30, respectively.

### The proposed severity estimation model

**Fig. 2:**
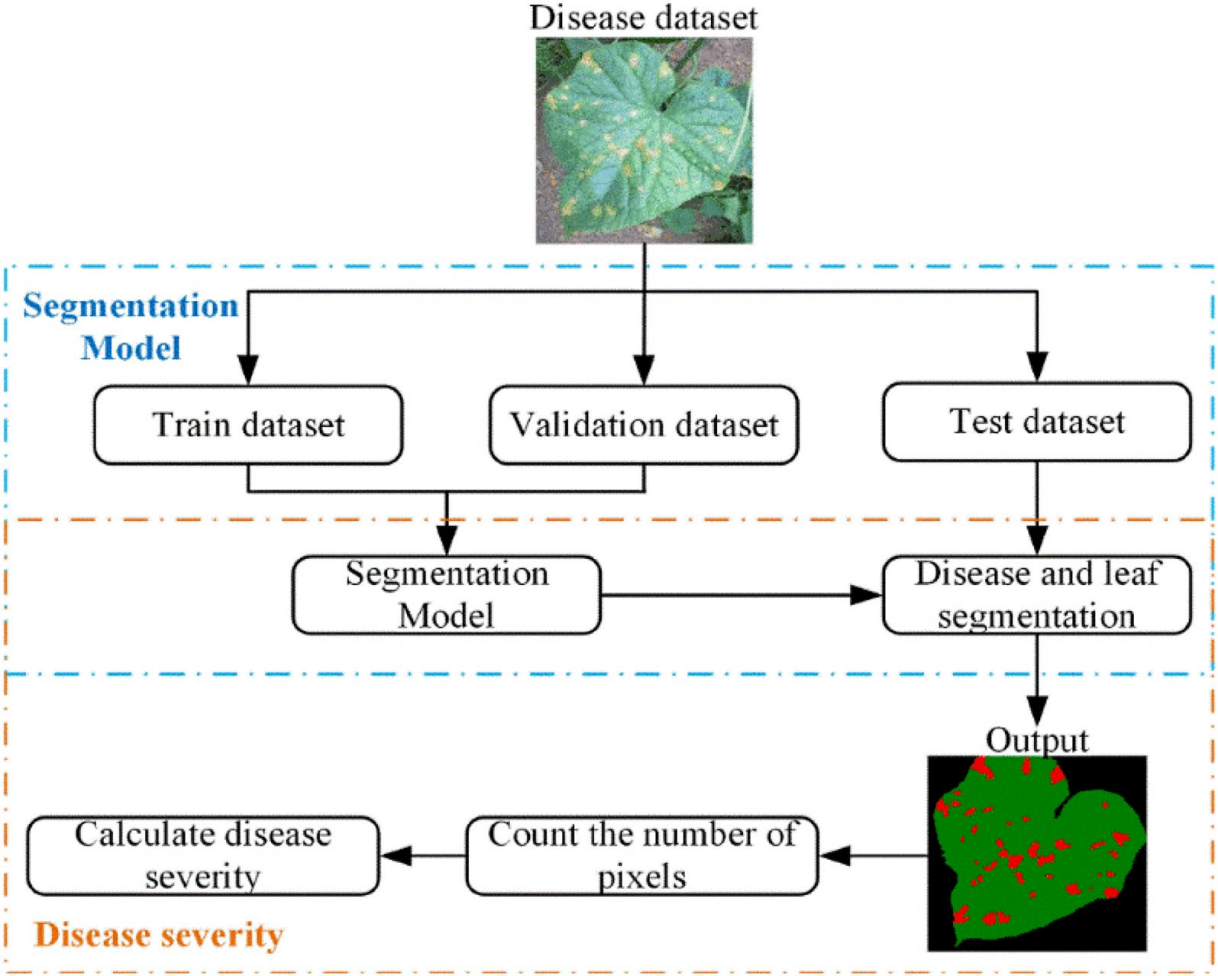
Overall flowchart of severity estimation

This study aims to automatically calculate the disease severity using semantic segmentation to assign category labels to each image pixel. The pipeline of the proposed severity estimation model is shown in Fig. 2. The training dataset is used to train the proposed segmentation model, and the validation dataset is applied to tune the hyperparameters of the model and perform an initial assessment of the model accuracy. The performance of the proposed segmentation model is evaluated and compared over the test dataset. Finally, the numbers of the healthy leaf pixels and the lesion pixels are separately counted in the segmentation results, achieving the disease severity according to Eq. (1).

### Segmentation network

**Fig. 3:**
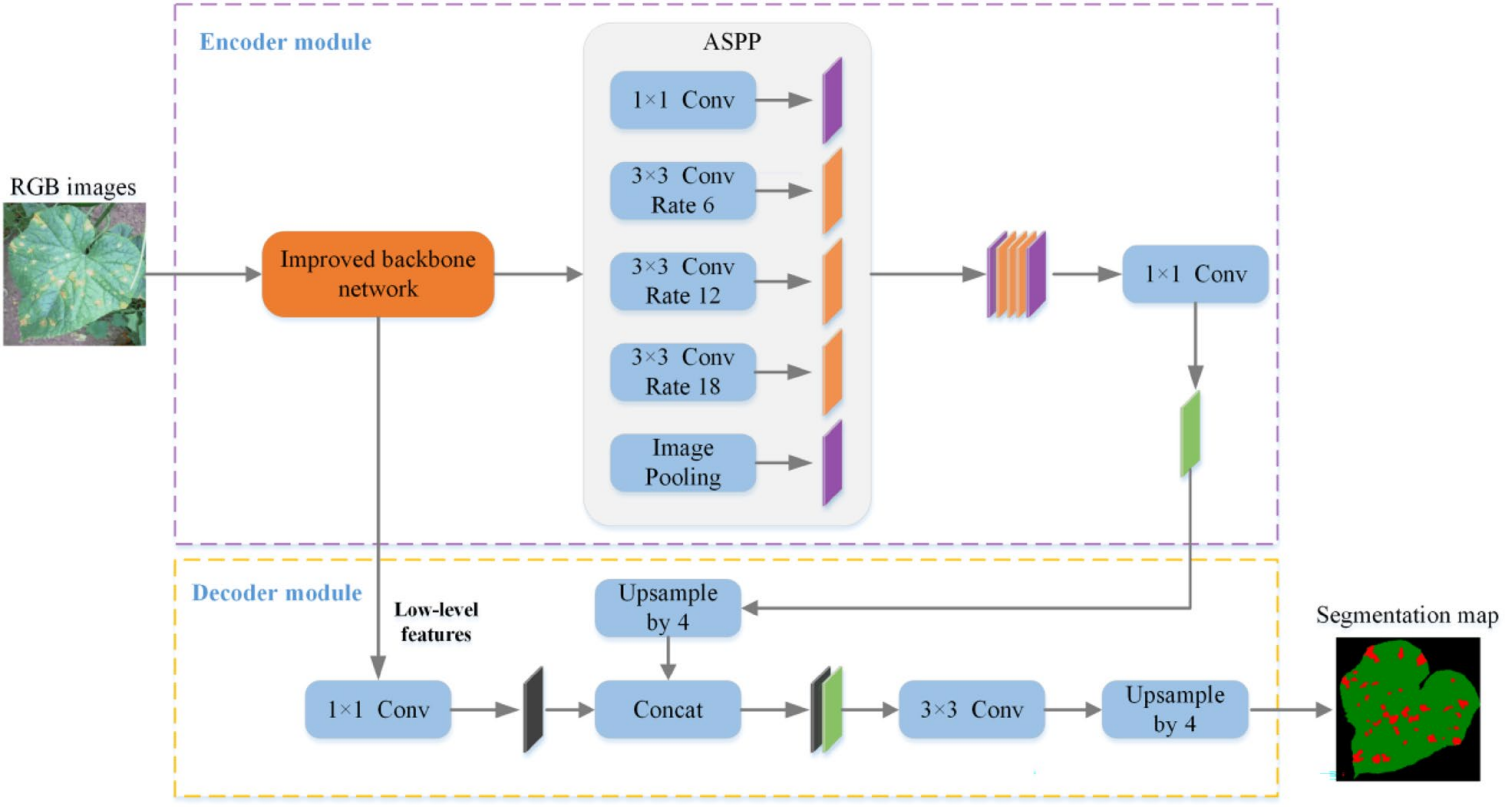
Block diagram of proposed model for disease image segmentation. The orange block indicates the improved backbone, the blue block indicates operations of convolution, pooling, concatenation and upsampling

Previous studies have shown that DeepLab V3 + can achieve remarkable segmentation results for lesions [21] and plants [18]. Consequently, it is optimized in this study as the benchmark model for severity estimation. The block diagram of the proposed segmentation model is shown in Fig. 3. It consists of two main blocks: an Encoder and a Decoder.

**Fig. 4:**
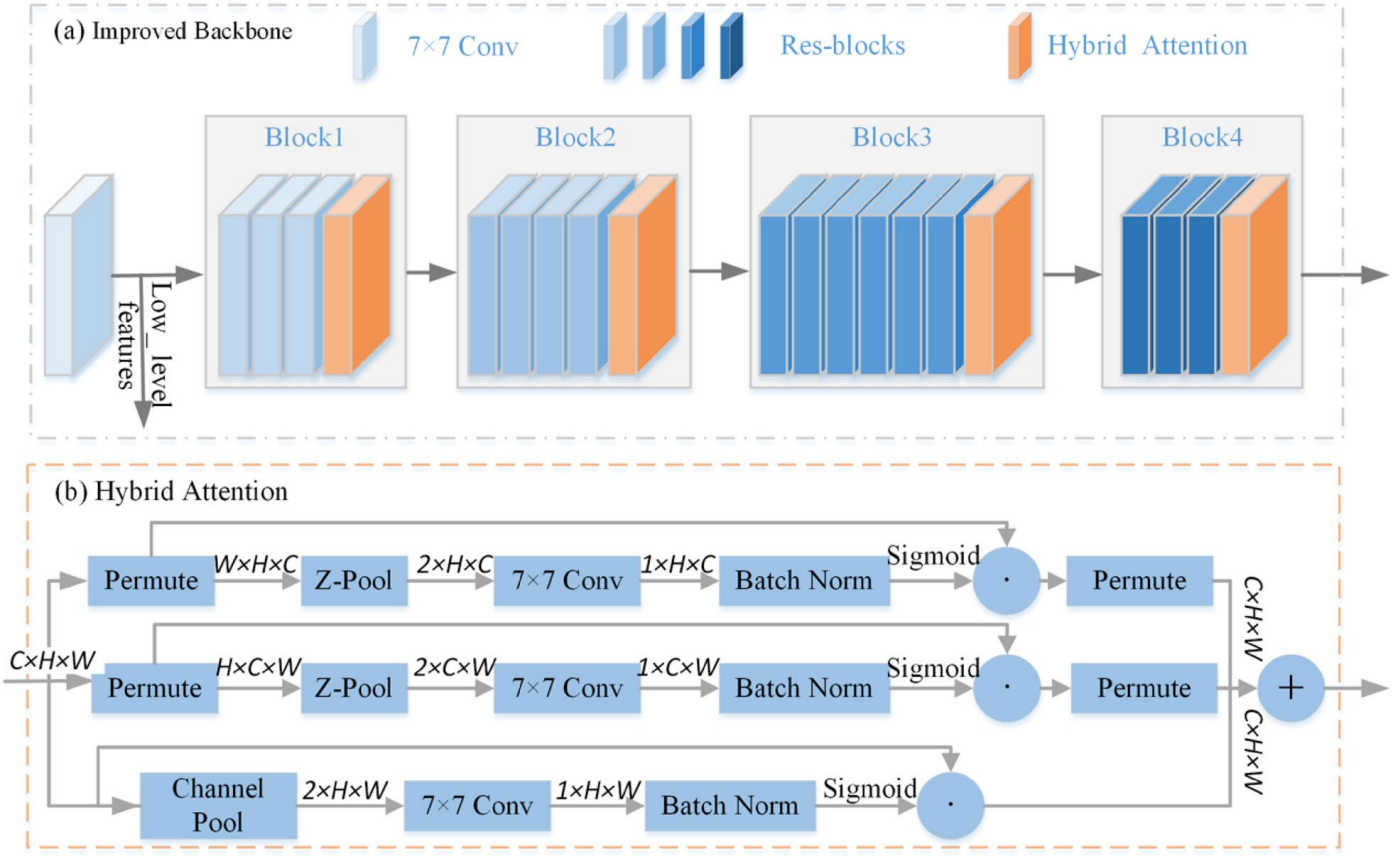
Block diagram of the improved backbone network for disease image segmentation **a** represents the improved backbone blocks and **b** shows the three branches of hybrid attention

In order to efficiently extract disease features in a complex context, the Encoder consists of improved the backbone network (Fig. 4) and the Atrous Space Pyramid Pool (ASPP). The improved backbone network uses a hybrid attention optimized ResNet50. ASPP performs parallel operations of Atrous convolution with multiple dilation rates and pooling. Three $3\times 3$ convolutions are performed with dilation rates of 6, 12 and 18, respectively. Different dilation rates can expand the receptive field and improve the localization detection accuracy without losing resolution. This operation condenses the features extracted by the improved backbone network into multi-scale contextual semantic information.

The following operations are performed in the Decoder: the output features of the Encoder are first bilinearly upsampled by a factor of 4, followed by concatenation with the low-level features from the improved backbone in the channel dimension. In order to reduce the number of channels of low-level features, a $1\times 1$ convolution is performed on the low-level features before applying the concatenation, followed by a $3\times 3$ convolution operation to refine the features. Finally, a simple bilinear upsampling by a factor of 4 is applied to produce the final semantic segmentation results.

### Attention mechanism

Disease images collected in field conditions have complex backgrounds. At the same time, the severity estimation requires neglecting the influence of background information and focusing on the segmentation of healthy leaves and lesions. The attention mechanism can select the key features of the current task from a large amount of available information. Introducing the attention mechanism into the network structure can help the model to facilitate feature selection and reduce recognition errors, thus improving the segmentation performance [24, 25].

In order to improve the performance of proposed model, a backbone based on residual blocks and a hybrid attention mechanism is proposed in this study. Figure 4 illustrates the architecture of the improved backbone network. It is a feature extractor optimized by the Hybrid Attention (HA) mechanism. The ResNet50 is adopted as the benchmark block [26] as the issues caused by the gradients vanishing/exploding are to be considered when training a deeper neural network. The hybrid attention mechanism [27] is added after each of the four residual blocks, as shown in Fig. 4(a). It helps the network to capture key internal representations of the image. In Fig. 4(b), the hybrid attention introduces cross-dimension interaction by dedicating three branches to capture dependencies between the $(C,H),(C,W)$ and $(H,W)$ dimensions of the input tensor. The first two branches can extract channel attention, while the last branch extracts spatial attention. In addition, hybrid attention is formed by summing and averaging both channel and spatial attention of the three branches. This hybrid attention can emphasize the importance of each dimensional feature in the tensor and extract richer feature information related to the target, which improves the segmentation accuracy [27].

Specifically, an input tensor $X \in R^{C \times H \times W}$ is delivered to each of the three branches in the hybrid attention module. In the first branch, the input X is rotated by 90˚ anticlockwise along the H axis, i.e., the permute operation in Fig. 4(b). When this rotated tensor passes through the Z-pool, it continues through the 7 × 7 standard convolutional layer and passes through the batch normalization layer in turn. The attention weight is generated by the sigmoid activation layer (σ) and is applied to the rotated tensor. Finally, it is rotated 90˚ clockwise along the H axis to retain the original input shape of X. Similarly, in the second branch, X is rotated by 90° anticlockwise along the W axis. The remaining operations are similar to the first branch. The final branch is similar to the Convolutional Block Attention Module (CBAM) [28], used to build spatial attention. The Z-pool layer is responsible for reducing the zeroth dimension of the tensor to two by concatenating the average pooled and max pooled features across that dimension, as it is calculated according to Eq. (2). This operation allows the layer to preserve a rich representation of the actual tensor while simultaneously shrinking its depth to make further computation lightweight.

2 $${\text{Z - pool(X) = [MaxPool}}_{{{\text{0d}}}} {\text{(X), AvgPool}}_{{{\text{0d}}}} {\text{ (X)]}}$$

where 0d is the 0th-dimension across which the max and average pooling operations take place. For instance, the Z-Pool of a tensor of shape $(C \times H \times W)$ results in a tensor of shape $(2 \times H \times W)$.

### Transfer learning

**Fig. 5:**
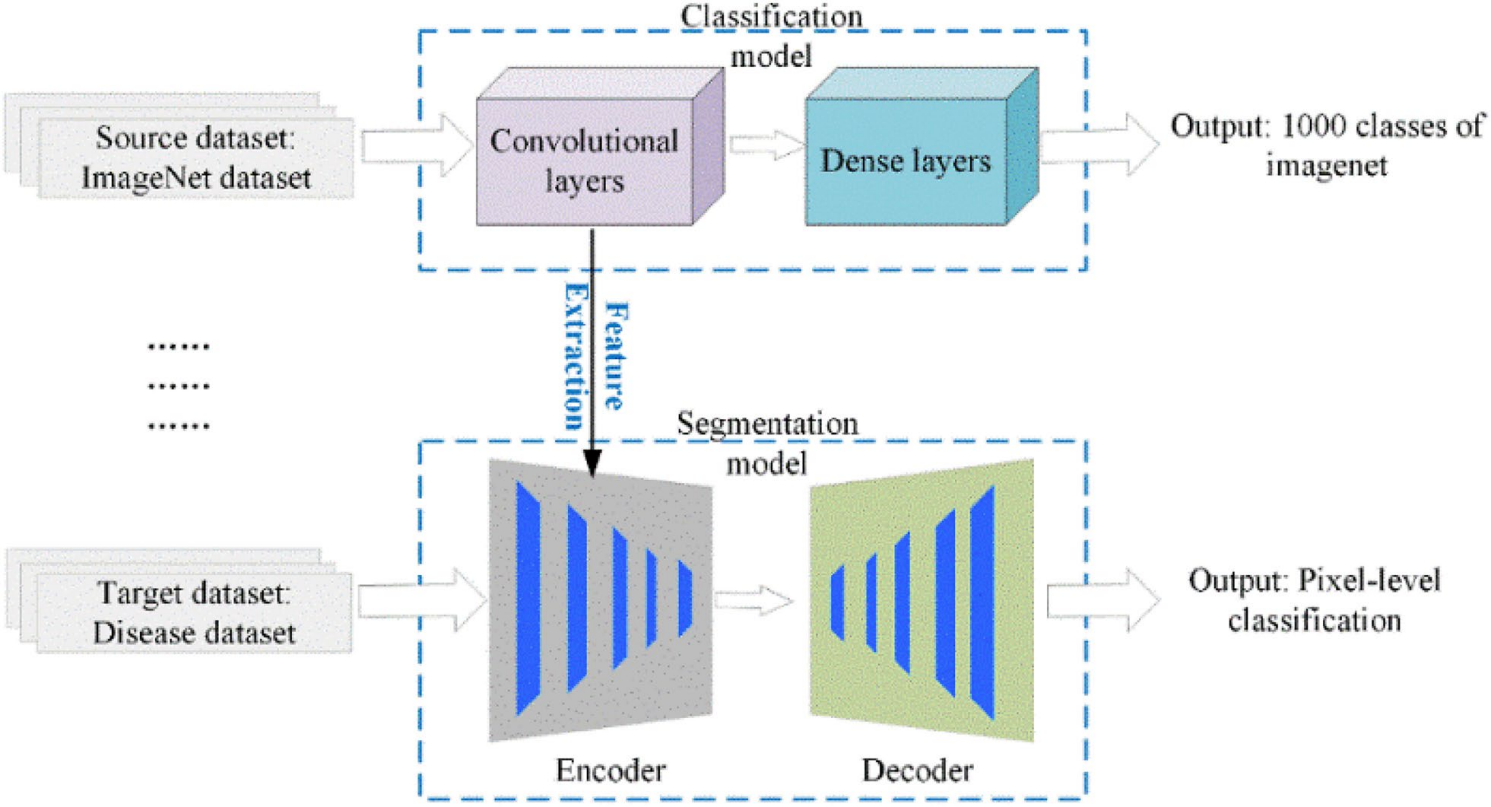
Transfer learning for optimizing the learning process

Manual image labeling is often time-consuming and labor-intensive, especially when dealing with applications such as lesion segmentation. In general, the number of images is not large enough for training a model from scratch. Transfer Learning (TL), which uses millions of labeled images for pre-training [22], provides a solution for the issue. TL can adapt well to the task by retraining on a relatively small dataset. Therefore, the TL strategy can reduce human efforts on image labeling. In the application of severity estimation, the proposed backbone is pre-trained on the ImageNet. It is then retrained on the disease dataset built in this study (Fig. 5).

### Loss function

In this study, the disease dataset has a much smaller number of pixels in the background and the lesion category than in the healthy leaf category. The frequency difference between the three categories can lead to an unbalanced effect on the training while ignoring the importance of diseased pixels. Therefore, the weighted cross-entropy loss function is used in the experiments to reduce this unbalanced effect. The weights of each category are calculated according to the median frequency balance of [29], as shown in Eq. (3). The final weights used for the dataset are 1.0000, 0.2286, and 3.4532 for the background, healthy leaf, and lesion.

3 $${W}_{m}=\frac{median\_fre}{{fre}_{m}},$$

where ${fre}_{m}$ represents the frequency of occurrences of pixels of class m divided by the total number of pixels in any image containing this class, and $median\_fre$ represents the median of these frequencies for all the classes.

The weights of the three categories are applied to the pixel-wise cross-entropy loss function:

4 $$L=-\frac{1}{N}{\sum }_{n=1}^{N}{\sum }_{m=0}^{M}{W}_{m}*{y}_{n,m}log {p}_{n,m}$$

where N is the number of observations, M is the number of target categories excluding the background, ${W}_{m}$ is the weight for class m, y is an indicator if a class label is correctly classified for observation n, and p is the predicted probability of observation n being of class m.

### Experimental operation environment

The proposed model is implemented based on the Python deep learning libraries of PyTorch and trained with an NVIDIA Quadro P2000 GPU (5 GB). Transfer learning is used to accelerate convergence. The encoder parameters are initialized with the pre-trained weights on the ImageNet, while the other parameters are initialized from a Gaussian distribution [26]. The Stochastic Gradient Descent (SGD), having a momentum of 0.9, is used in the training process. The parameters are tuned as many times as the device allows. The initial learning rate is 0.007, which varies in a Poly manner [23]. The maximum number of epochs used for training is 300, while the batch size is 8. The L2 regularization with a weight decay of 0.0001 is applied to the parameters to prevent overfitting.

### Performance evaluation

Since this study involved disease segmentation and severity estimation, the assessment was divided into segmentation and estimation. The segmentation results obtained by the proposed model are evaluated using the Intersection over Union (IoU) [30], Precision, Recall and F-1 score, which can be calculated from the confusion matrix [2].

The overall model performance is evaluated using the Accuracy (Acc), the Mean Intersection over Union (MIoU), and the Frequency Weighted Intersection over Union (FWIoU) [24]. Acc (Eq. (5)) represents the ratio of correctly segmented pixels over the total pixels. MIoU (Eq. (6)) is the mean IoU value of the background, leaf and lesion categories. FWIoU (Eq. (7)) sets the weights according to the frequency of each class, and FWIoU is a more objective representation of the model’s overall performance. The overall performance is computed as:

5 $$Acc=\frac{{\sum }_{i=0}^{M}{p}_{ii}}{{\sum }_{i=0}^{M}{\sum }_{j=0}^{M}{p}_{ij}}$$

6 $$MIoU=\frac{1}{M+1}\sum_{i=0}^{M}\frac{{p}_{ii}}{{\sum }_{j=0}^{M}{p}_{ij}+{\sum }_{j=0}^{M}{{p}_{ji}-p}_{ii}}$$

7 $$FWIoU=\frac{1}{{\sum }_{i=0}^{M}{\sum }_{j=0}^{M}{p}_{ij}}\sum_{i=0}^{M}\frac{{p}_{ii}}{{\sum }_{j=0}^{M}{p}_{ij}+{\sum }_{j=0}^{M}{{p}_{ji}-p}_{ii}}$$

where ${p}_{ii}$ denotes the number of pixels of class i that are predicted as class i, and ${p}_{ij}$ denotes the number of pixels of class i that are predicted as class j.

The accuracy of the severity estimation is evaluated using the coefficient of determination (R^2^) and the Root Mean Square Error (RMSE) [17].

## Results and discussion

This section presents the results of the disease severity estimation, including the quantitative evaluation results of the pixel-wise segmentation and the severity calculation. The methods are trained and evaluated using the “Experimental operation environment” settings. The contributions of TL and HA on the proposed model are first investigated. Different backbones and attention mechanisms are then evaluated. Finally, the proposed model is compared with the state-of-the-art models.

### Comparison of estimation results of hybrid attention and transfer learning

An ablation study is performed to evaluate the contributions of the significant components to the model. DeepLab V3 + with ResNet50 as the backbone network is the baseline model. A comparison between the improved models is performed. The obtained results are shown in Tables 1 and 2.

**Table 1:** Results of the proposed method on the test dataset

Methods	Baseline	TL	HA	Acc (%)	MIoU (%)	FWIoU (%)	*R*^2^	RMSE
Baseline	√			88.24	67.22	79.16	0.7754	3.0093
Baseline + TL	√	√		92.75	74.13	86.82	0.8477	2.7365
Baseline + HA	√		√	94.24	78.16	89.52	0.9042	2.8739
Baseline + TL + HA (Proposed)	√	√	√	95.64	81.23	91.89	0.9578	1.1385

**Table 2:** Results of the proposed method for each category on the disease dataset

Methods	IoU (%)			F-1 (%)		
	Background	Leaf	Lesion	Background	Leaf	Lesion
Baseline	69.67	85.96	47.35	82.12	91.67	64.27
Baseline + TL	83.13	89.77	49.50	90.79	94.61	66.23
Baseline + HA	86.97	91.81	55.70	93.03	95.73	71.55
Baseline + TL + HA (Proposed)	89.01	94.14	60.55	94.18	96.98	75.43

The results show that the baseline model achieves an Acc of 88.24% (Table 1). When TL is used, the performance of the Baseline + TL is further improved. More precisely, the Acc value increases to 92.75%. Similarly, the MIoU and FWIoU values are improved by 6.91–7.66%, respectively. The IoU of leaf category and lesion category increases by 3.81–2.15% after using TL, respectively. It can be clearly seen from Table 1 that TL can significantly improve the results of severity estimation, with R^2^ reaching 0.8477.

Baseline + HA achieves the most significant improvement of 10.94% in MIoU, followed by 10.36% in FWIoU and 6% in Acc (Table 1). Simultaneously, the corresponding improvement in IoU and F-1 for all the three categories is obtained, with the most significant improvement of 8.35% in IoU for the lesions (Table 2). The R^2^ value of the Baseline + HA increases from 0.7754 to 0.9042, achieving an improvement of 0.1288 (Table 1). It can also be seen from Table 1 that the HA reaches an improvement of 7.1% for MIoU based on the use of transfer learning methods. It is important to mention that HA significantly effects on lesion category, with an improvement of almost 11.05% in IoU (Table 2). The results show that HA significantly promotes disease segmentation, especially for the lesion category. In other words, HA extracts detailed disease features through the cross-dimensional interactions of space and channels, enhancing the disease segmentation profile, and improving the severity estimates (R^2^ = 0.9042).

**Fig. 6:**
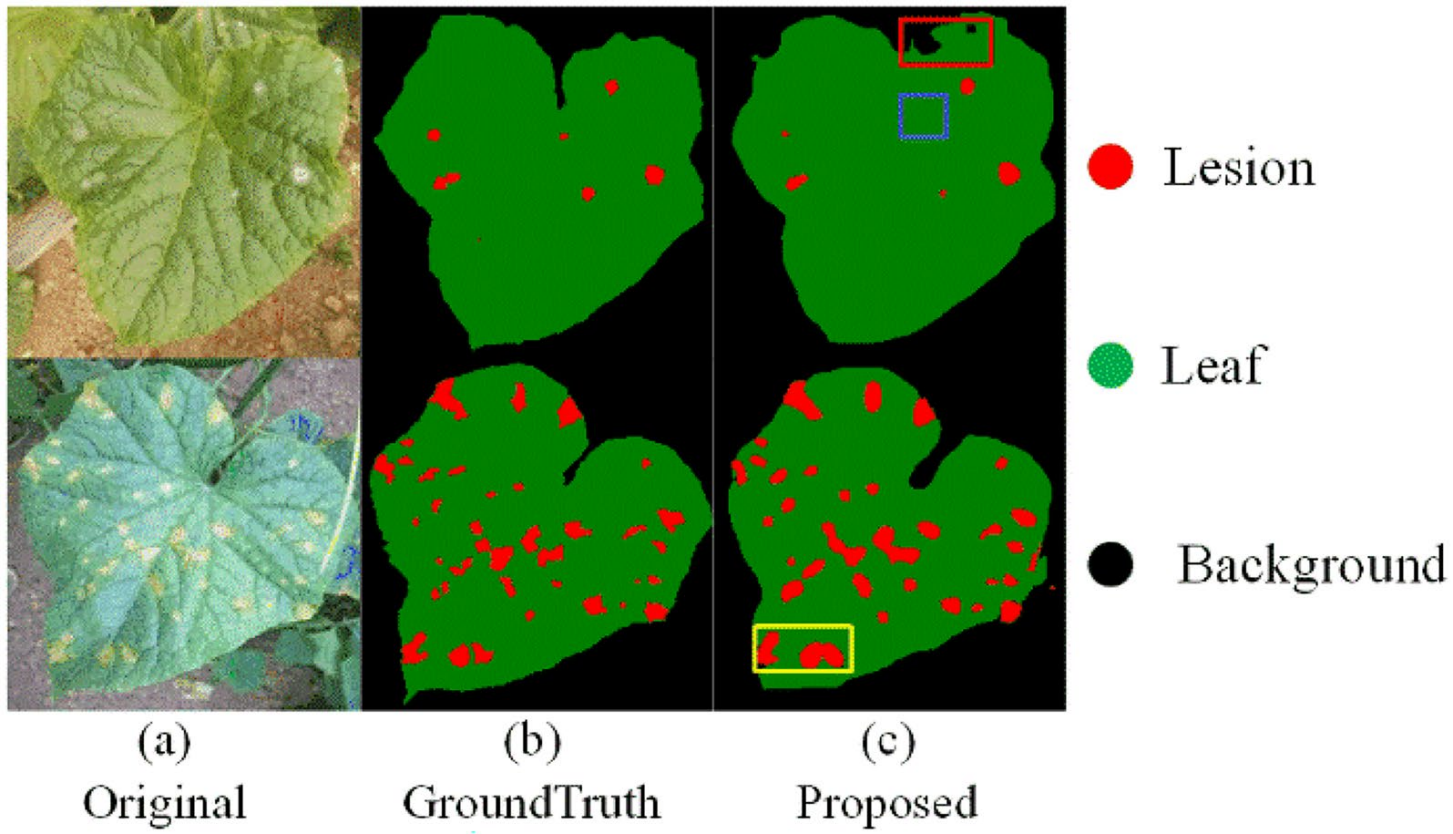
Samples of segmentation errors. Different colored boxes indicate different types of errors

Figure 6 shows some segmentation errors. The first error type is caused by the other cucumber leaves with similar colors and unclear borders in the images obtained under the field conditions (red box in Fig. 6). The second error type is related to the misclassification of the lesion pixels (blue box in Fig. 6). Some of the target lesions are small and similar to the leaf pixels under strong illumination. The third error type is the over-segmentation of the lesions (yellow box in Fig. 6). The edges of the lesion area are unclear, such as the downy mildew having irregular faded greenish-yellow lesions on the leaves. The boundaries of the non-greenish yellow halo part are challenging, leading to similar errors in the model [5]. However, the overall described segmented images closely follow the artificial segmentation criteria [19]. Finally, for cucumber leaf disease images collected in real scenarios, the proposed method is able to accurately achieve automatic estimation of disease severity by segmenting lesions and leaves.

### Comparison of estimation results of different backbones

**Fig. 7:**
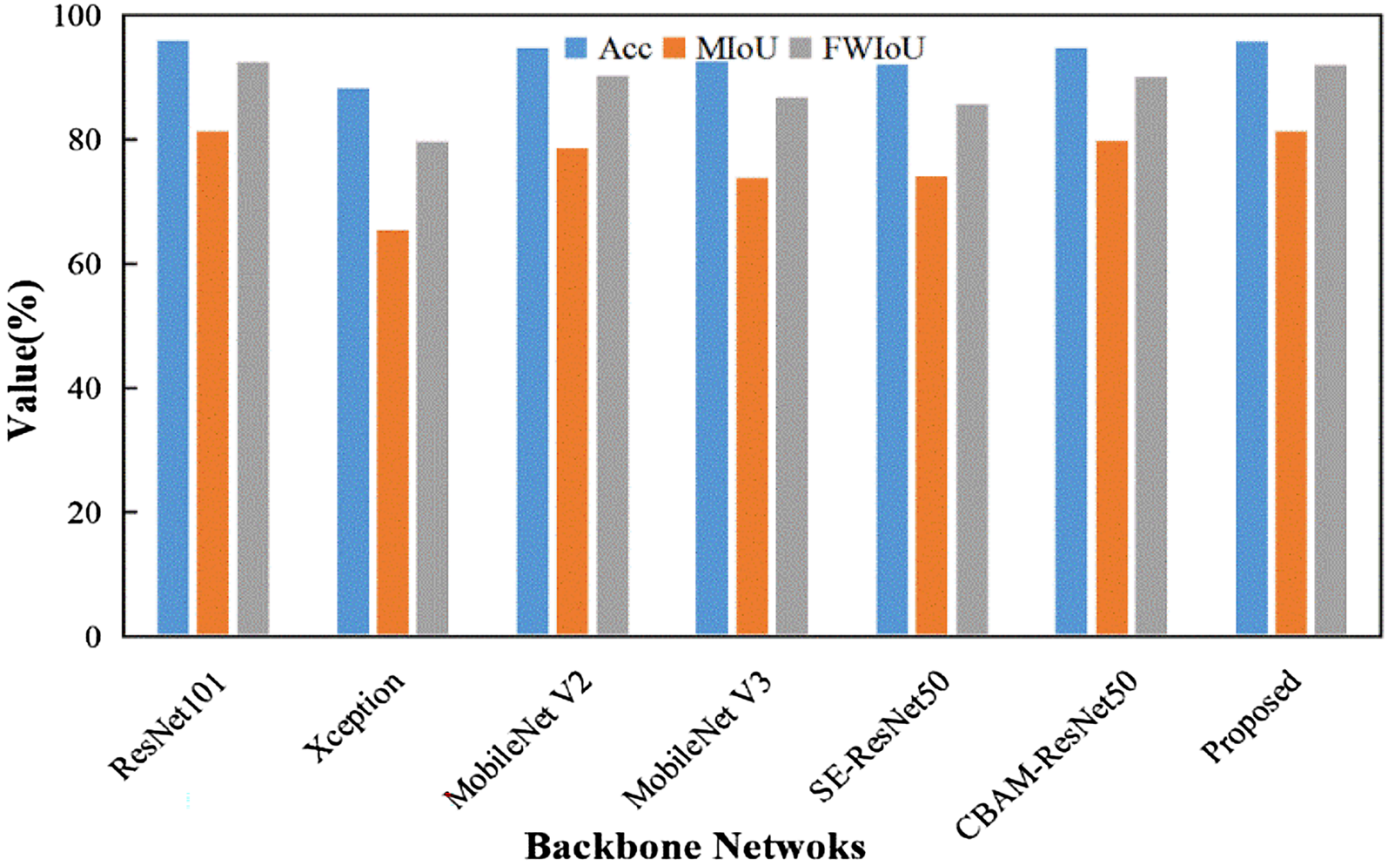
Segmentation performance of the different backbone networks

In order to further validate the efficiency of the proposed backbone for disease segmentation, several backbone networks based on the DeepLab V3 + segmentation framework are compared: Xception [23], MobileNet V2 [31], MobileNet V3 [32], ResNet101 [26], SE-ResNet50 (Squeeze and Expansion attention-optimized ResNet50) [33] and CBAM-ResNet50 (CBAM attention-optimized ResNet50)[28]. The segmentation results of the models based on different backbones are shown in Fig. 7 and Table 3.

**Table 3:** Segmentation results of the different backbones

Backbone networks	Class	Precision (%)	Recall (%)	F-1 (%)	IoU (%)
ResNet101	Background	95.63	95.1	95.36	91.14
	Leaf	97.27	96.62	96.94	94.07
	Lesion	65.72	84.32	73.87	58.56
Xception	Background	90.97	76.54	83.13	71.13
	Leaf	89.87	93.79	91.79	84.82
	Lesion	43.61	83.82	57.37	40.23
MobilenetV2	Background	95.36	91.1	93.18	87.23
	Leaf	95.69	96.57	96.13	92.55
	Lesion	62.45	84.62	71.86	56.08
MobilenetV3	Background	92.28	89.16	90.70	82.98
	Leaf	94.46	94.68	94.57	89.69
	Lesion	55.74	78.55	65.21	48.37
SE-ResNet50	Background	89.52	89.00	89.26	80.6
	Leaf	94.38	93.98	94.18	89.01
	Lesion	62.82	76.14	68.84	52.48
CBAM-ResNet50	Background	95.72	90.49	93.03	86.97
	Leaf	95.18	97.00	96.08	92.45
	Lesion	68.72	82.1	74.82	59.76
Proposed	Background	94.71	93.66	94.18	89.01
	Leaf	97.01	96.96	96.98	94.14
	Lesion	71.03	80.41	75.43	60.55

The results show that the ResNet101 achieves a very close performance to the proposed backbone, where both are better than the CBAM-ResNet50, MobileNet V2, ResNet50, MobileNet V3 and SE-ResNet50 (Fig. 7). Xception is the worst performing backbone on the disease dataset. Table 3 shows the same results as the ones presented in [21]. Specifically, all the backbone networks have higher performance for the background category than for the leaf and lesion categories. Since this study aims at calculating the disease severity, the segmentation accuracy for both the leaf and the lesion should be guaranteed. According to the evaluation metrics of the pixel-wise classification, the proposed backbone has the highest performance for segmenting leaf and lesion categories (Table 3). The proposed backbone leads to significant improvements in the leaf and lesion categories compared with other backbones, especially in the lesion category, with F-1 improving by 0.61–18.06% and IoU improving by 0.79–20.32% (Table 3).

The impacts of several attentional mechanisms on the disease segmentation are also studied. An interesting finding is that the performance of SE-ResNet50 is slightly decreased. However, SE attention has a specific effect on the lesion’s segmentation, resulting in a corresponding improvement in both F-1 and IoU (Table 3). Both CBAM-ResNet50 and the proposed model outperform the Baseline + TL (Tables 1 and 2) in terms of overall performance. Their performance is improved by 5.6–7.1% on MIoU, respectively. It can also be seen from Table 3 that the inclusion of the attention mechanism endorses the model to focus on the lesion features, which results in improving the segmentation performance on the category lesion.

**Fig. 8:**
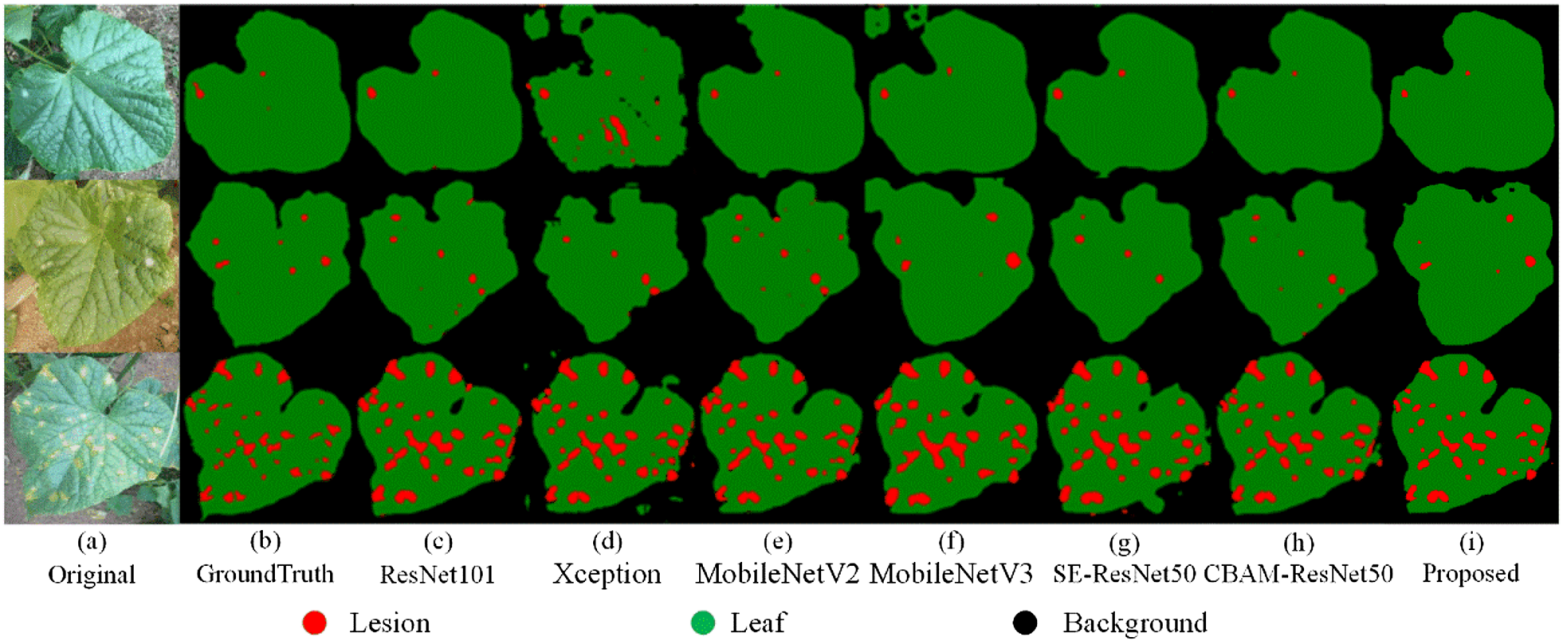
Segmentation results from different backbones

Figure 8 shows the segmentation results of different backbones. Considering the segmentation results of leaves and lesions, the segmentation of CBAM-ResNet50 is the second after the proposed backbone. It incorporates attention mapping in two separate dimensions [28], and achieves accurate segmentation of the lesions. However, CBAM-ResNet50 does not perform well in segmenting the background boundaries close to the target leaves (Fig. 8h). Due to the deeper level of ResNet101, many high level features can be extracted, resulting in better disease segmentation performances. Nevertheless, there also exists the same misclassifications for lesions (Fig. 8c). In addition, the segmentation models based on MobileNet V2 and MobileNet V3 show different degrees of leaf over-segmentation (Fig. 8e and Fig. 8f). SE-ResNet50 learns the correlation between channels [33]. Therefore, there is some improvement in lesion segmentation (Fig. 8g). Finally, the segmentation model of Xception has difficulties in segmenting the whole leaf (Fig. 8d). The segmentation ability for complex backgrounds is poor. The proposed backbone accurately extracts lesion and leaf features by capturing cross-dimensional interactions [27], inhibiting the effects of backgrounds noise and efficiently segmenting leaves and lesions.

**Fig. 9:**
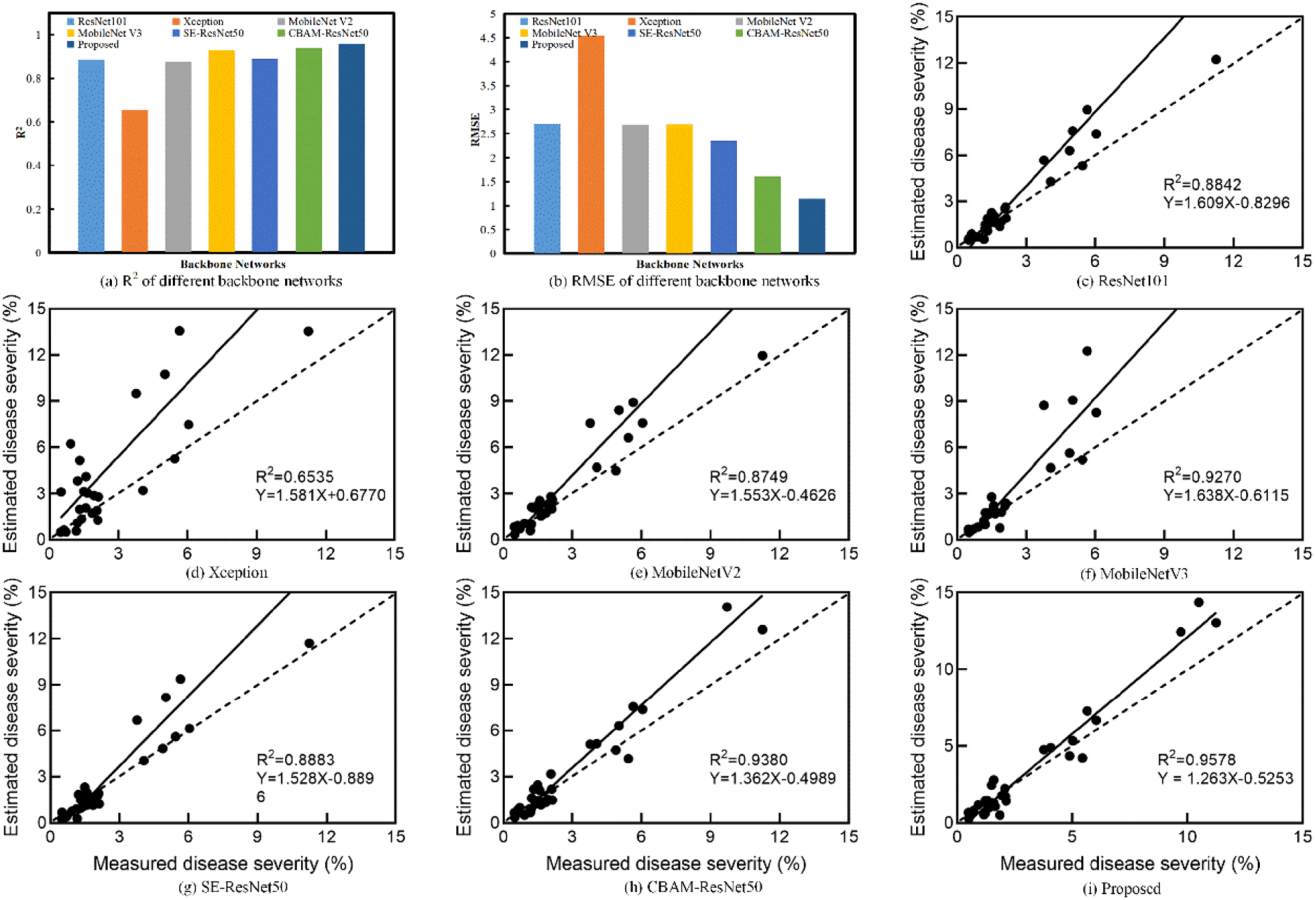
Severity estimation results based on different backbone networks. **a**, **b** R^2^ and RMSE of different backbone networks. **c**–**i** regression results of different backbone networks, where the dashed line denotes the 1:1 line

Similarly, this study aims to evaluate the backbone efficiency for disease severity estimation. Thus, a linear fit of the severity, based on the comparison of different backbone segmentation models to the actual severity, is drawn on the test dataset (Fig. 9). The results show that the proposed method achieves the highest R^2^ and the lowest RMSE in the severity estimation (Fig. 9a and Fig. 9b). The R^2^ of different backbones are generally higher than 0.87, and the RMSE values are lower than 2.7, except for Xception. The results show that the severity estimation models based on pixel-wise classification can reasonably estimate the disease severity [3]. Moreover, most disease severity is overestimated when estimating the disease severity by the segmentation results of the semantic segmentation models (Fig. 9) [3, 19]. This result may be explained by the fact that the models misclassify the leaf pixels as category lesion.

### Comparison of estimation results of the state-of-the-art models

Many studies have shown that deep learning-based methods are able to achieve better performance than the shallow machine learning-based methods in image classification tasks [17, 20]. In order to verify the efficiency of the proposed model for severity estimation, a comparison is performed with the state-of-the-art semantic segmentation models: FCN [30], Unet [34], SegNet [29] and DeepLab V3 + .

**Fig. 10:**
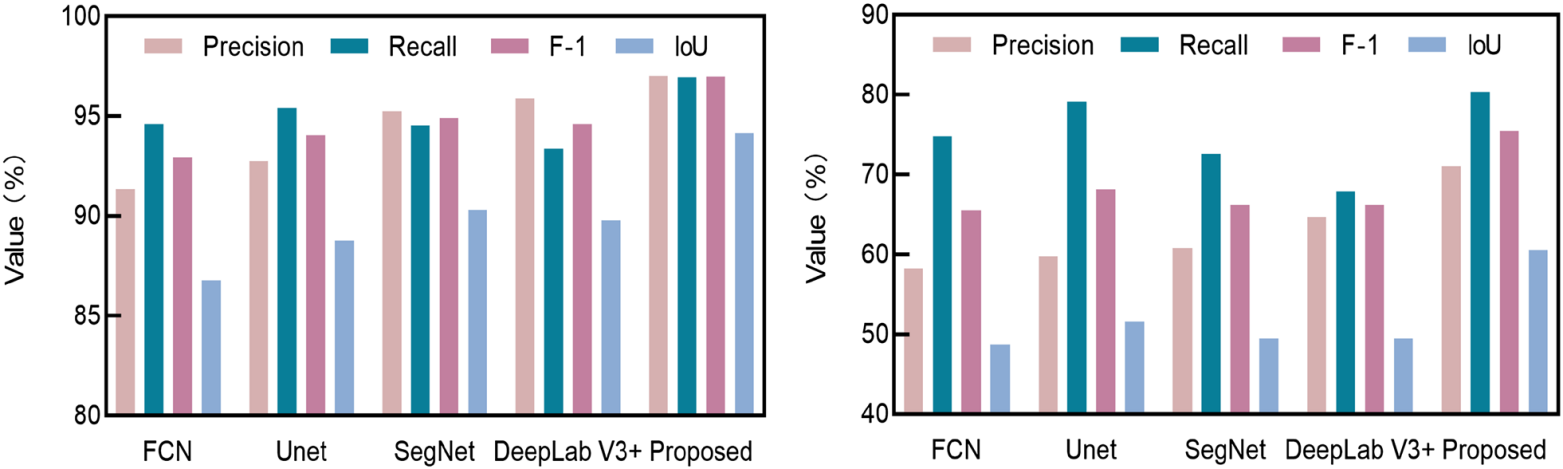
Performances of the segmentation models on **a** category leaf, and **b** category lesion

Table 4 presents the Acc, MIoU, and FWIoU for all the models on the test dataset. Figure 10 shows the evaluation metrics of the models for both the leaf and the lesion. The results confirm that the proposed model performs better than the other methods. It outperforms FCN by 11.17%, Unet by 8.04%, SegNet by 6.64% and DeepLab V3 + by 7.1% on MIoU (Table 4). In particular, for the lesion category (Fig. 10), the proposed model outperforms FCN by 9.92%, Unet by 7.34%, SegNet by 9.21% and DeepLab V3 + by 9.2% on F-1. For the leaf category, the proposed model leads to an improvement of at least 3.84%, over these four models on IoU.

**Table 4:** Comparison results of the different segmentation methods

Methods	Acc (%)	MIoU (%)	FWIoU (%)
FCN	90.05	70.06	82.2
Unet	91.71	73.19	84.98
SegNet	93.09	74.59	87.44
DeepLab V3 +	92.75	74.13	86.82
Proposed	95.64	81.23	91.89

Table 4 and Fig. 10 validate the efficiency of the proposed model for leaf and lesion semantic segmentation. Nonetheless, the complex lesion boundaries and the small targets result in low performance of all the segmentation algorithms for lesion segmentation (Fig. 10 b) [2].

**Fig. 11:**
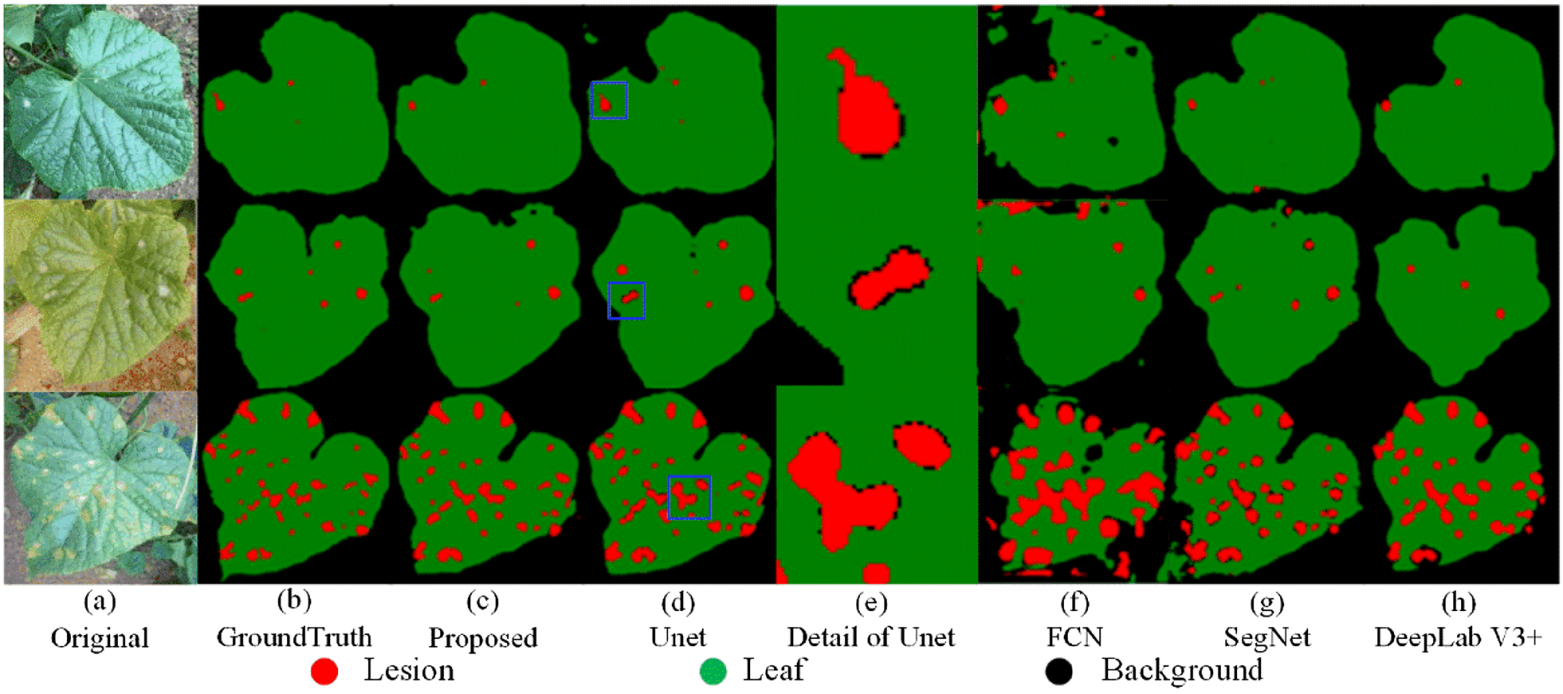
Segmentation results of different models

The segmentation results of all the models are shown in Fig. 11. It can be clearly seen that FCN misses many details when facing the complex backgrounds and the small target [24]. FCN is not sensitive enough to the details of the image and misses the semantic information between pixels. DeepLab V3 + uses an ASPP module to encode the multi-scale contextual information and suppress backgrounds interference [35]. The segmentation results of SegNet are relatively better than those of FCN and DeepLab V3 + . This may be explained by the fact that SegNet uses the pooling indices from the high-resolution features for segmentation, which will reserve helpful detailed information. Figure 11e shows the detailed information of the Unet segmentation results with incorrect lesion border segmentation [36]. It shows that the lesion and the leaf are incorrectly segmented into the backgrounds. Although the proposed model uses the same segmentation framework as DeepLab V3 + , it extracts the hybrid attention of spatial and channel interactions, thus capturing more features of the disease images [27]. Consequently, the proposed method significantly improves the segmentation of leaves and lesions, and efficiently reduces the occurrence of under-segmentation.

**Fig. 12:**
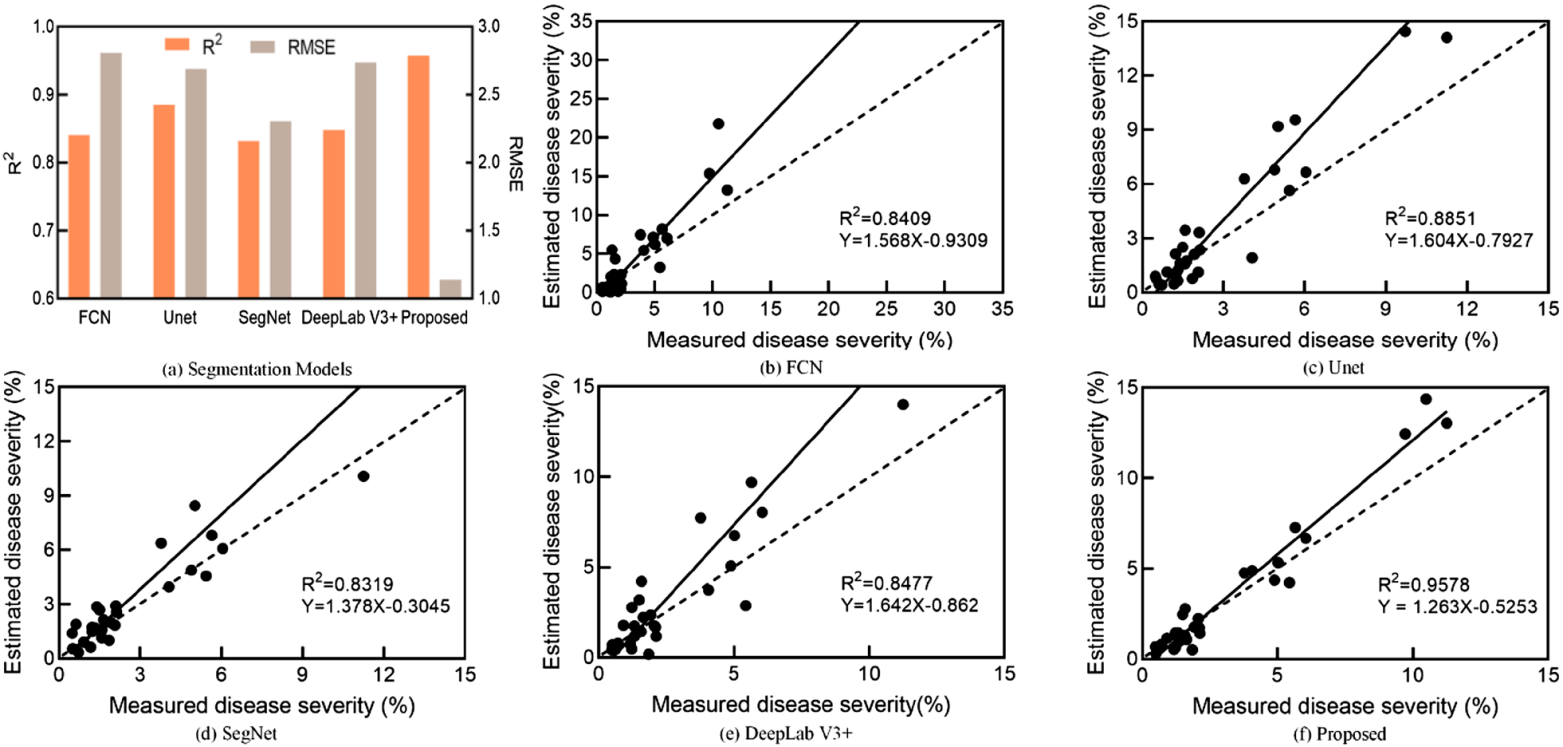
Severity estimation results based on semantic segmentation networks. **a** Summary results. **b**–**e** regression results of different segmentation models, where the dashed line denotes the 1:1 line

The reliability of the semantic segmentation models is evaluated according to the severity estimation results (Fig. 12). The results show that the R^2^ values of all the models are generally greater than 0.83 (Fig. 12a), ranging from 0.83 (FCN) to 0.96 (proposed method). FCN and SegNet yield relatively poor estimation. DeepLab V3 + and Unet achieve slightly better severity estimations, with R^2^ values of 0.8477 and 0.8851, respectively. The estimated severity by the proposed model has a good agreement with the actual severity values, with a highest R^2^ of 0.9578 and a lowest RMSE of 1.1385 (Fig. 12f). This result depends on the accurate segmentation of the lesions and leaves by the proposed method. In general, most of the models tend to overestimate the severity [19], leading to higher estimation errors.

It can be seen from Fig. 9 and Fig. 12 that the semantic segmentation model estimates a higher severity for images with high true severity. Figure 8 and Fig. 11 show that the severity overestimated due to the large area of the lesions in the severely diseased leaves. Many small lesions also adhere to each other, leading to inaccurate segmentation of healthy leaves and lesions by the segmentation model. This is also consistent with the visual judgement of the naked eye [2].

In summary, the proposed method is relatively unaffected by the complex background. It is able to accurately segment lesions and leaves from cucumber disease images collected in field conditions. Moreover, this method does not require multiple stages to segment leaves and lesions, which can save computing resources. In addition, the model achieves high estimation accuracy in severity estimation. The proposed method can be generalized to segment other crops in future work.

## Conclusion

This study develops an integrated method for cucumber downy mildew and powdery mildew severity assessment based on the attention-optimized DeepLab V3 + . The proposed method achieves accurate disease segmentation in field conditions, by obtaining segmentation IoU equal to 94.14% and 60.55% for leaves and lesions, respectively. For the disease severity estimation, the RMSE and R^2^ are 1.1385 and 0.9578, respectively. The previous problems of time-consumption and low accuracy of visual disease severity estimation are solved, helping researchers to quickly study the disease resistance phenotype of cucumber.

In addition, the residual network optimized by hybrid attention is used as the backbone for DeepLab V3 + . The hybrid attention can capture the cross-latitude interaction between the space and the channel, significantly refining disease segmentation and improving the severity estimation accuracy. The common knowledge of ResNet50 is transferred from ImageNet. Transfer learning allows the use of generic features and segmentation networks to be trained on limited datasets, thus improving the accuracy of disease segmentation on small datasets.

Furthermore, a comparative analysis of 4 semantic segmentation models (FCN, Unet, SegNet and DeepLab V3 +) and 6 backbones (ResNet101, Xception, MobileNet V2, MobileNet V3 and attention-ResNet50) is performed. The experimental results show that the proposed method outperforms other models in severity estimation, and the R^2^ is improved by almost 0.318.

For cucumber disease images with one leaf, a relatively accurate model for severity estimation is developed. It is one of the directions to further enrich the construction of multi-crop and multi-leaf severity estimation models based on deep learning. In future work, we aim to generalize the model to other vegetables and diseases. In addition, the disease severity estimation model trained in this study should be further developed and settled to mobile devices to promote field management of the cucumber growth process.

## Data Availability

The datasets used and/or analysed during the current study are available from the corresponding author on reasonable request.

## Abbreviations

DL: Deep learning
CNN: Convolutional neural networks
ASPP: Atrous space pyramid pool
HA: Hybrid attention
CBAM: Convolutional block attention module
TL: Transfer learning
SGD: Stochastic gradient descent
IoU: Intersection over union
MIoU: Mean intersection over union
FWIoU: Frequency weighted intersection over union
RMSE: Root mean square error
SE: Squeeze and expansion
SE-ResNet50: Squeeze and Expansion attention-optimized ResNet50
CBAM-ResNet50: CBAM attention-optimized ResNet50
FCN: Fully convolutional networks
